# Supplementary material for: Resolving colistin resistance and heteroresistance in Enterobacter species
Source: Nat Commun. 2023 Jan 10;14:140. doi: 10.1038/s41467-022-35717-0 (PMC9832134; doi:10.1038/s41467-022-35717-0)
Supplement: Supplementary file 1 — Reporting Summary [file 41467_2022_35717_MOESM1_ESM.pdf]

nature portfolio

Corresponding author(s): Trinad Chakraborty

Last updated by author(s): Nov 28, 2022

Reporting Summary

Nature Portfolio wishes to improve the reproducibility of the work that we publish. This form provides structure for consistency and transparency in reporting. For further information on Nature Portfolio policies, see our Editorial Policies and the Editorial Policy Checklist.

Statistics

For all statistical analyses, confirm that the following items are present in the figure legend, table legend, main text, or Methods section.

n/a	Confirmed	
☐	☒	The exact sample size (*n*) for each experimental group/condition, given as a discrete number and unit of measurement
☐	☒	A statement on whether measurements were taken from distinct samples or whether the same sample was measured repeatedly
☐	☒	The statistical test(s) used AND whether they are one- or two-sided<br>*Only common tests should be described solely by name; describe more complex techniques in the Methods section.*
☒	☐	A description of all covariates tested
☒	☐	A description of any assumptions or corrections, such as tests of normality and adjustment for multiple comparisons
☐	☒	A full description of the statistical parameters including central tendency (e.g. means) or other basic estimates (e.g. regression coefficient) AND variation (e.g. standard deviation) or associated estimates of uncertainty (e.g. confidence intervals)
☐	☒	For null hypothesis testing, the test statistic (e.g. *F*, *t*, *r*) with confidence intervals, effect sizes, degrees of freedom and *P* value noted<br>*Give P values as exact values whenever suitable.*
☒	☐	For Bayesian analysis, information on the choice of priors and Markov chain Monte Carlo settings
☒	☐	For hierarchical and complex designs, identification of the appropriate level for tests and full reporting of outcomes
☒	☐	Estimates of effect sizes (e.g. Cohen's *d*, Pearson's *r*), indicating how they were calculated

Our web collection on statistics for biologists contains articles on many of the points above.

Software and code

Policy information about availability of computer code

Data collection	The NCBI genome data was downloaded by eutilities v2.0
Data analysis	Trimomatic v0.36, Porechop v0.2.4, FastQC v0.11.9, Unicycler v0.4.8, JSpecies v1.2.1, Enveomics package v1.9.0, genome-to-genome distance calculator (GGDC) using formula-2 (GGDChttps://ggdc.dsmz.de), RhierBAPS v1.0.1, Scoary v1.6.16, MASH v2.1.1, iTOL v4, Easyfig v2.1, PlasmidFinder v2.0.1, snippy v4.3.6, Ugene v1.32.0, Resistance Gene Identifier' v5.05.5m Swiss-model server, PROCHECK, HADDOCK, PyMOL v2.0, Xcalibur v3.1, ImageJ v1.53e

For manuscripts utilizing custom algorithms or software that are central to the research but not yet described in published literature, software must be made available to editors and reviewers. We strongly encourage code deposition in a community repository (e.g. GitHub). See the Nature Portfolio guidelines for submitting code & software for further information.

Data

Policy information about availability of data

All manuscripts must include a data availability statement. This statement should provide the following information, where applicable:

- Accession codes, unique identifiers, or web links for publicly available datasets
- A description of any restrictions on data availability
- For clinical datasets or third party data, please ensure that the statement adheres to our policy

All the sequencing data is publicly available under the BioProject accession number PRJNA622426

Fluorescence microscopy raw images are submitted as a dataset on Zonedo (https://zenodo.org/record/7405690#.Y49a5qjMKUl) with DOI: 10.5281/zenodo.7405690

The publicly available repositories used are
https://www.ncbi.nlm.nih.gov/genome/?term=Enterobacter
https://www.ncbi.nlm.nih.gov/sra/?term=Enterobacter
https://www.ncbi.nlm.nih.gov/pathogens/antimicrobial-resistance/AMRFinder/
CARD database (v3.0.3)
https://www.ncbi.nlm.nih.gov/pathogens/isolates#/search
http://www.wwpdb.org

Human research participants

Policy information about studies involving human research participants and Sex and Gender in Research.

Reporting on sex and gender	Not applicable
Population characteristics	Not applicable
Recruitment	Not applicable
Ethics oversight	Not applicable

Note that full information on the approval of the study protocol must also be provided in the manuscript.

Field-specific reporting

Please select the one below that is the best fit for your research. If you are not sure, read the appropriate sections before making your selection.

☒ Life sciences ☐ Behavioural & social sciences ☐ Ecological, evolutionary & environmental sciences

For a reference copy of the document with all sections, see nature.com/documents/nr-reporting-summary-flat.pdf

Life sciences study design

All studies must disclose on these points even when the disclosure is negative.

Sample size	The sample size was selected based on previous experience, the published literature, feasibility, and the level of variability between replicates. No statistical methods were used to predetermine sample size.
Data exclusions	No data was excluded
Replication	Minimum inhibitory concentration (MIC), mass-spectrometry, and real-time PCR were repeated for three times by independent biological experiment to determine the variation caused by the 'heteroresistance' phenomenon. The fluorescence microscopy experiments were repeated for two-times.
Randomization	No randomization was carried out for 165 isolates studied and all the isolates were studied for their genomes and MIC. For mass spectrometry and fluorescence microscopy isolates best representing species (based on the phylogenomic placement or that showed MIC and heteroresistance average to species) were considered for analysis/experiments.
Blinding	Blinding was relevant to this study involving all bacterial genomics and bioinformatics.

Reporting for specific materials, systems and methods

We require information from authors about some types of materials, experimental systems and methods used in many studies. Here, indicate whether each material, system or method listed is relevant to your study. If you are not sure if a list item applies to your research, read the appropriate section before selecting a response.

Materials & experimental systems

n/a	Involved in the study
☒	☐ Antibodies
☒	☐ Eukaryotic cell lines
☒	☐ Palaeontology and archaeology
☒	☐ Animals and other organisms
☒	☐ Clinical data
☒	☐ Dual use research of concern

Methods

n/a	Involved in the study
☒	☐ ChIP-seq
☒	☐ Flow cytometry
☒	☐ MRI-based neuroimaging
