# Supplementary material for: Resolving colistin resistance and heteroresistance in Enterobacter species
Source: Nat Commun. 2023 Jan 10;14:140. doi: 10.1038/s41467-022-35717-0 (PMC9832134; doi:10.1038/s41467-022-35717-0)
Supplement: Supplementary file 2 — Supplementary Information [file 41467_2022_35717_MOESM2_ESM.docx]

## Supplementary Material

## Journal: Nature Communications

## Title: Resolving colistin resistance and heteroresistance in *Enterobacter* species

**Authors:** Swapnil Prakash Doijad^1,2#^, Nicolas Gisch^3#^, Renate Frantz^1,2^, Bajarang Vasant Kumbhar^4^, Jane Falgenhauer^1,2^, Can Imirzalioglu^1,2^, Linda Falgenhauer^1,2,5^, Alexander Mischnik^1,6^, Jan Rupp^1,6^, Michael Behnke^1,7^, Michael Buhl^1,8,9,10^, Simone Eisenbeis^1,9^, Petra Gastmeier^1,7^, Hanna Gölz^1,11^, Georg Alexander Häcker,^1,11^ Nadja Käding^1,6^, Winfried V. Kern^1,12^, Axel Kola^1,7^, Evelyn Kramme^6,7^, Silke Peter^1,8^, Anna M. Rohde^1,7^, Harald Seifert^1,13^, Evelina Tacconelli^1,9^, Maria J.G.T. Vehreschild^1,14,15^, Sarah V. Walker^1,13^, Janine Zweigner^1,13^, Dominik Schwudke^1,3,16^, on behalf of the DZIF R-Net Study Group^$^ and Trinad Chakraborty^1,2*^

**Address for correspondence:** Prof. Dr. Trinad Chakraborty, Institute of Medical Microbiology, Justus-Liebig University, German Center for Infection Research (DZIF), Partner Site Gießen-Marburg-Langen, Campus Gießen, Justus-Liebig University, Gießen 35394, Germany.

E-mail: Trinad.Chakraborty@mikrobio.med.uni-giessen.de

# Supplementary Tables and Figures

## Supplementary Table 1: ‘Overall Genomic Relatedness Index’ (OGRI) of type strains of *Enterobacter hormaechei, Enterobacter hoffmannii, Enterobacter oharae, Enterobacter steigerwaltii* and *Enterobacter xiangfangensis*. While *Enterobacter* *xiangfangensis* is a valid taxonomical species^1^, this species was proposed as a subspecies of the *Enterobacter* *hormaechei*^2^*,* resulting taxonomic confusion. To get more clarity in the identification of the species, we reassessed their taxonomic allocations by the approach recommended by the International Committee on Systematics of Prokaryotes (ICSP)^3^, such as JSpecies (1-way-ANI), Enveomics (2-way-ANI), and genome-to-genome distance calculator (GGDC). In brief, we determined the OGRI based on the ANI as calculated by JSpecies v1.2.1 (1-way-ANI). The 1-way-ANI between *E.* *hormaechei* ATCC 49162^T^ and *E.* *xiangfangensis* LMG 27195^T^ showed an ANI of 94.7%, which was slightly less than the recommended cut-off of 95-96%, indicating their separate species candidature. Considering the borderline values, we also used additional tools recommended by ICSP such as the Enveomics package that calculates the 2-way-ANI and GGDC2.1 (formula 2) to calculate the ‘Digital DNA-DNA hybridization’ score calculated by (GGDC). The 2-way-ANI uses the same cut-off of ANI (>95-96%), while the recommended cut-off for the GGDC is 70%. The 2-way-ANI and GGDC values for *E.* *hormaechei* and *E.* *xiangfangensis* were less than the recommended cut-offs to define a species, showing they are two distinct species. Subsequently, *steigerwaltii* DSM 16691^T^, *oharae* DSM 16687^T^ and *hoffmannii* DSM 14563^T^ are all subspecies of the *E. xiangfangensis*. Nevertheless, as there are no clear criteria to define the subspecies, we limited the description of the isolates only to the species level.

|  | *E. hormaechei* ATCC 49162^T^ (MKEQ01) | | | *E.* *hoffmannii* DSM 14563^T^ (CP0171838) | | | *E. oharae* DSM 16687^T^  (CP017180) | | | *E.* *steigerwaltii* DSM 16691^T^ (CP017179) | | | *E.* *xiangfangensis* LMG 27195^T^ (CP017183) | | |
| --- | --- | --- | --- | --- | --- | --- | --- | --- | --- | --- | --- | --- | --- | --- | --- |
|  | 1-way-ANI | 2-way-ANI | GGDC | 1-way-ANI | 2-way-ANI | GGDC | 1-way-ANI | 2-way-ANI | GGDC | 1-way-ANI | 2-way-ANI | GGDC | 1-way-ANI | 2-way-ANI | GGDC |
| *E. hormaechei* ATCC 49162^T^ (MKEQ01) | --- | --- | --- | 94.3 | 93.8 | 58 | 95 | 94.7 | 63 | 94.9 | 94.5 | 62 | 94.7 | 94.2 | 60 |
| *E. hoffmannii* DSM 14563^T^ (CP0171838) | 94.4 | 93.8 | 58 | --- | --- | --- | 95.8 | 95.5 | 67 | 95.8 | 95.5 | 67 | 95.9 | 95.5 | 67 |
| *E. oharae* DSM 16687^T^ (CP017180) | 95.1 | 94.7 | 62 | 95.8 | 95.5 | 67 | --- | --- | --- | 97.6 | 97.5 | 81 | 97.2 | 96.9 | 76 |
| *E. steigerwaltii* DSM 16691^T^ (CP017179) | 94.8 | 94.5 | 62 | 95.7 | 95.5 | 67 | 97.5 | 97.6 | 81 | --- | --- | --- | 96.9 | 96.9 | 76 |
| *E. xiangfangensis* LMG 27195^T^ (CP017183) | 94.7 | 94.3 | 60 | 95.8 | 95.5 | 67 | 97.1 | 96.9 | 76 | 97.1 | 96.9 | 76 | --- | --- | --- |

## Supplementary Table 2: Distribution of *E. xiangfangensis* and other *Enterobacter* species isolates from bloodstream infection and colonization.

| **Site** | **Total No. of Isolates** | **bloodstream isolates** | | |  | **colonization isolates** | | |
| --- | --- | --- | --- | --- | --- | --- | --- | --- |
|  |  | **Total no. of isolates** | ***E. xiangfangensis* (% of total blood isolates)** | **other species (% of total blood isolates)** |  | **Total** | ***E. xiangfangensis***  **(% of total colonization isolates)** | **other species**  **(% of total colonization isolates)** |
| Berlin | 30 | 3 | 3 (3.1) | 0 (0.0) |  | 27 | 21 (30.4) | 6 (8.7) |
| Cologne | 37 | 22 | 16 (16.7) | 6 (6.3) |  | 15 | 6 (8.7) | 9 (13.0) |
| Freiburg | 30 | 20 | 13 (13.5) | 7 (7.3) |  | 10 | 7 (10.1) | 3 (4.3) |
| Giessen | 21 | 17 | 12 (12.5) | 5 (5.2) |  | 4 | 0 (0.0) | 4 (5.8) |
| Lübeck | 34 | 27 | 19 (19.8) | 8 (8.3) |  | 7 | 4 (5.8) | 3 (4.3) |
| Tübingen | 13 | 7 | 4 (4.2) | 3 (3.1) |  | 6 | 3 (4.3) | 3 (4.3) |
| **Total** | 165 | 96 | 67 (69.8) | 29 (30.2) |  | 69 | 41 (59.4) | 28 (40.6) |

## Supplementary Table 3: Geographical location-wise distribution of *E. xiangfangensis* and other *Enterobacter* species.

| **Geographical location** | **Total number of isolates in NCBI** | ***E. xiangfangensis* (%)** | **Other *Enterobacter* species (%)** |
| --- | --- | --- | --- |
| USA | 951 | 64.6 | 35.4 |
| United Kingdom | 574 | 79.8 | 20.2 |
| China | 179 | 50.8 | 49.2 |
| Australia | 127 | 82.7 | 17.3 |
| Netherlands | 121 | 86.0 | 14.0 |
| France | 53 | 52.8 | 47.2 |
| Singapore | 53 | 35.8 | 64.2 |
| Germany | 46 | 65.2 | 34.8 |
| India | 39 | 53.8 | 46.2 |
| Nepal | 39 | 97.4 | 2.6 |
| Colombia | 36 | 75.0 | 25.0 |
| Spain | 27 | 70.4 | 29.6 |
| South Africa | 20 | 60.0 | 40.0 |
| Greece | 18 | 100.0 | 0.0 |
| Ireland | 17 | 100.0 | 0.0 |
| Brazil | 16 | 68.8 | 31.3 |
| Malaysia | 16 | 25.0 | 75.0 |
| Japan | 14 | 21.4 | 78.6 |
| Belgium | 13 | 100.0 | 0.0 |
| Lebanon | 13 | 92.3 | 7.7 |
| Viet Nam | 12 | 66.7 | 33.3 |
| Pakistan | 10 | 70.0 | 30.0 |
| Philippines | 10 | 50.0 | 50.0 |
| Romania | 10 | 100.0 | 0.0 |
| Argentina | 9 | 88.9 | 11.1 |
| Croatia | 9 | 100.0 | 0.0 |
| Italy | 8 | 62.5 | 37.5 |
| Nigeria | 8 | 75.0 | 25.0 |
| Serbia | 8 | 100.0 | 0.0 |
| Canada | 7 | 14.3 | 85.7 |
| Taiwan | 7 | 85.7 | 14.3 |
| Togo | 7 | 42.9 | 57.1 |
| Austria | 6 | 83.3 | 16.7 |
| Tunisia | 6 | 66.7 | 33.3 |
| Turkey | 6 | 100.0 | 0.0 |
| Kuwait | 5 | 40.0 | 60.0 |
| Mexico | 5 | 80.0 | 20.0 |
| South Korea | 5 | 40.0 | 60.0 |
| Thailand | 5 | 80.0 | 20.0 |
| Morocco | 4 | 100.0 | 0.0 |
| Norway | 4 | 25.0 | 75.0 |
| Guatemala | 3 | 66.7 | 33.3 |
| Peru | 3 | 66.7 | 33.3 |
| Portugal | 3 | 100.0 | 0.0 |
| Russia | 3 | 100.0 | 0.0 |
| Venezuela | 3 | 33.3 | 66.7 |
| Bangladesh | 2 | 50.0 | 50.0 |
| Bolivia | 2 | 100.0 | 0.0 |
| Cameroon | 2 | 0.0 | 100.0 |
| Czech Republic | 2 | 100.0 | 0.0 |
| Denmark | 2 | 0.0 | 100.0 |
| Gaza Strip | 2 | 100.0 | 0.0 |
| Ghana | 2 | 0.0 | 100.0 |
| Jamaica | 2 | 0.0 | 100.0 |
| Nicaragua | 2 | 0.0 | 100.0 |
| Puerto Rico | 2 | 50.0 | 50.0 |
| Ecuador | 1 | 100.0 | 0.0 |
| Egypt | 1 | 100.0 | 0.0 |
| Hungary | 1 | 100.0 | 0.0 |
| Israel | 1 | 100.0 | 0.0 |
| Jordan | 1 | 100.0 | 0.0 |
| Kenya | 1 | 0.0 | 100.0 |
| Mauritius | 1 | 100.0 | 0.0 |
| Oman | 1 | 0.0 | 100.0 |
| Poland | 1 | 100.0 | 0.0 |
| Switzerland | 1 | 0.0 | 100.0 |
| Tanzania | 1 | 0.0 | 100.0 |

## Supplementary Table 4: Prevalence of *mcr-1* to *-9* among the members of the *Enterobacteriaceae*. Tables A and B depict data from the isolates obtained from clinical and environmental/other sources, respectively. This data is derived from the curated genome database of the NCBI pathogen detection browser (<https://www.ncbi.nlm.nih.gov/pathogens/>). The blue and red bar's length next to the numbers in each cell depicts the relative number of isolates or percentages, respectively. Among the *mcr-9*-positive *Enterobacter* species (clinical and environmental/other sources), 20.9% of the isolates were *E. xiangfangensis* (Lineage-wise: 21.2% L-1, 19.0% L-2, 22.7% L-3 and 16.7% L-4 ), 20% *E. asburiae*, 22.7% *E. kobei*, 11.4% *E. roggenkampii* and 4.5% *E. cloacae.*

## Supplementary Table 5: Comparison of modified lipid-A to minimum inhibitory concentration (MIC_LB_) and frequency of heteroresistance (HRF). Isolates analyzed for lipid-A modifications and those encoding *arnBCADTEF* were examined for MIC_LB_, % of heteroresistant cells, and % of live cells. Isolates were grown at identical conditions. The % of lipid-A modified with L-Ara4N were positively associated (Pearson’s correlation coefficient, r = 0.88 and 0.73) with the % of heteroresistance CFUs (at 8 and 32 mg/L), and % live cells (r = 0.86) with intact membranes after the colistin treatment (64 mg/L for 15 min). This data suggest colistin heteroresistance is directly associated with the amount of modified lipid A. (nd: not done)

| **Species** | **arn** | **strain** | **MIC µg/ml** | **% live cells** | **% modified Lipid A** | **% HRF** | |
| --- | --- | --- | --- | --- | --- | --- | --- |
|  |  |  |  |  |  | **8 mg/L** | **32 mg/L** |
| *E. asburiae* | + | BK5433 | 128-256 | 50.98 ± 1.77 | 23.10 ± 1.05 | 5.30 ± 1.77 | 1.20 ± 0.19 |
| *E. bugandensis* | + | RBG-17-0246-1 | 64-128 | 66.67 ± 4.43 | 41.87 ± 6.00 | 10.41 ± 2.45 | 1.63 ± 0.37 |
| *E. cancerogenus* | + | RPK-18-0479-1 | 64-128 | 10.84 ± 5.07 | 37.93 ± 7.23 | 1.78 ± 1.03 | 1.39 ± 1.15 |
| *E. chengduensis* | + | RBK-18-0141-1 | 64-128 | 65.83 ± 15.71 | 37.54 ± 2.42 | 12.65 ± 2.30 | 7.89 ± 1.16 |
| *E. cloacae* | + | RPK-18-0157-1 | 512->512 | 30.95 ± 10.98 | 29.37 ± 4.25 | 21.56 ± 2.87 | 11.12 ± 1.63 |
| *E. kobei* | + | BK6664 | 32 | 22.28 ± 5.91 | 06.45 ± 0.29 | 0.28 ± 0.08 | 0.11 ± 0 |
| *E. ludwigii* | + | F-8789 | 8-64 | 33.13 ± 1.49 | 06.10 ± 0.34 | 0.02 ± 0.01 | 0 |
| *E. wuhouensis* | - | F00728 | 4-6 | 1.12 ± 0 | 0 | 0 | 0 |
| *E. roggenkampii* | + | RPB-17-0516-2 | 64->128 | 18.32 ± 2.96 | 16.11 ± 0.56 | 2.17 ± 0.55 | 0.89 ± 0.17 |
| *E. mori* | + | RBL-17-0354-2 | 32-64 | 7.32 ± 0.25 | 05.67 ± 1.65 | 1.21 ± 0.24 | 0.54 ± 0.01 |
| *E. vonholyi (*Δ*phoQ)* | + | F-147 | 2-4 | 1.29 ± 0.57 | 3.20 ± 1.09 | 0 | 0 |
| *E. xiangfangensis* L-2 | **-** | RPT-16-0254-2 | 2-4 | 0.12 ± 0.01 | 0 | 0 | 0 |
| *E. xiangfangensis* L-3 | **-** | RBG-18-0378-1 | 2-4 | 0.45 ± 0.28 | 0 | 0 | 0 |
| *E. xiangfangensis* L-3 | **-** | RBF-18-0224-1 | 2-4 | 1.11 ± 1.03 | 0 | 0 | 0 |
| *E. xiangfangensis* L-4 | **-** | RBL-17-0298-1 | 2-4 | 0.64 ± 0 | 0 | 0 | 0 |
| *E. xiangfangensis* L-1 | + | BK4615 | 2-16 | 16.40 ± 1.77 | 0.10 ± 0.14 | 0.19 ± 0 | 0 |
| *E. xiangfangensis* L-1 | + | RPF-17-0260-1 | 4-6 | 12.40 ± 1.32 | 0.16 ± 0.28 | 0.02 ± 0 | 0 |
| *E. xiangfangensis* L-1 | + | RBL-17-0437-1 | 2-8 | 10.17 ± 3.30 | 0.37 ± 0 | 0.04 ± 0 | 0.01 ± 0 |
| *E. xiangfangensis* L-1 | + | RBG-18-0107-1 | 4-16 | 12.06 ± 6.78 | 5.76 ± 2.48 | 0.18 ± 0 | 0 |
| *E. xiangfangensis* L-1 | + | RBK-17-0394-1 | 2-32 | 11.82 ± 0.01 | 3.35 ± 2.09 | 0.74 ± 0.27 | 0.50 ± 0.15 |
| *E. xiangfangensis* L-1 | + | RBK-17-0394-1 pH5 | 256->512 | nd | 37.69 ± 4.89 | 35.41 ± 7.78 | 5.33 ± 0.29 |
| *E. xiangfangensis* L-1 | + | RBK-17-0394-1 pH9 | 2 | nd | 4.17 ± 3.69 | 0 | 0 |
| *E. xiangfangensis* L-1 | - | RBK-17-0394-1 Δ*arn* | 0.5 | 3.81 ± 2.35 | 0 | 0 | 0 |
| *E. xiangfangensis* L-1 | + | RBK-17-0394-1 Δ*phoPQ* | 2 | 4.68 ± 1.29 | 0 | 0 | 0 |
| *E. xiangfangensis* L-1 | + | RBK-17-0394-1 Δ*mgrB* | 128->256 | 85.51 ± 7.89 | 78.62 ± 2.26 | 49.67 ± 3.86 | 50.67 ± 3.09 |
| *E. xiangfangensis* L-1 | + | RBK-17-0394-1 Δ*pmrAB* | 2-32 | nd | 10.12 ± 1.08 | 0.52 ± 0.01 | 0.31 ± 0.00 |
| *E. xiangfangensis* L-1 | + | RBK-17-0394-1 Δ*arn* + *arn* | 8-16 | nd | nd | 1.15 ± 0.85 | 0.62 ± 0.25 |
| *E. xiangfangensis* L-1 | + | RBK-17-0394-1 Δ*mgrB* + *mgrB* | 4-16 | nd | nd | 0.34 ± 0.24 | 0.22 ± 0.09 |
| *E. xiangfangensis* L-1 | + | RBK-17-0394-1 Δ*phoPQ* + *phoPQ_394_* | 8-16 | nd | 1.61 ± 0.67 | 0.63 ± 0.09 | 0.14 ± 0.07 |
| *E. xiangfangensis* L-1 | + | RBK-17-0394-1 Δ*phoPQ* + *phoPQ_246_* | 128 | nd | 66.61 ± 5.74 | 42.33 ± 4.78 | 7.13 ± 2.75 |
| *E. xiangfangensis* L-1 | + | RBK-17-0394-1 Δ*phoPQ* + *_phoPQ516_* | 64-128 | nd | 0.68 ± 0.62 | 10.83 ± 1.43 | 1.05 ± 0.19 |
|  |  | Correlation coefficient (r) to lipid A modification | | 0.86 |  | 0.88 | 0.73 |

## Supplementary Table 6: Heteroresistance capabilities of *phoP-phoQ* hybrid complemented mutants. The hybrid complement mutants were created by combining *phoP* and *phoQ* genes either of *E. xiangfangensis* 394, *E. bugandensis* 246 and *E. roggenkampii* 516. Plasmids harbouring *phoP*-*phoQ* hybrids were transferred to Ex394Δ*phoPQ*. The control *E. coli* ATCC 25922 showed MIC_LB_ less than 2 and exhibited no heteroresistance frequency. MIC_LB_ values for colistin are in mg/L.

| **strains** | ***phoP*** | ***phoQ*** | **MIC**_LB_ **at pH5** | **MIC**_LB_ **at**  **pH7** | **MIC**_LB_ **at**  **pH9** | **8C HR%** | **32C HR%** |
| --- | --- | --- | --- | --- | --- | --- | --- |
| WT_394_ | 394 | 394 | 256-512 | 2-32 | 4-8 | 0.745 (± 0.27) | 0.505 (± 0.15) |
| Δ*phoPQ_394_* | - | - | 4 | 4 | 4-8 | 0 | 0 |
| Δ*phoPQ_394_* + *phoPQ_394_* | 394 | 394 | 256 | 16-32 | 4-8 | 0.63 (± 0.09) | 0.14 (± 0.07) |
| Δ*phoPQ_394_* + *phoPQ_246_* | 246 | 246 | 256-512 | 128 | 4-8 | 42.33 (± 4.78) | 7.13 (± 2.75) |
| Δ*phoPQ_394_* + *phoPQ_516_* | 516 | 516 | 256-512 | 64-128 | 4-8 | 10.83 (± 1.43) | 1.05 (± 0.19) |
| Δ*phoPQ_394_* + *phoP_246_Q*_394_ | 246 | 394 | 256-512 | 16-32 | 4-8 | 1.20 (± 0.60) | 0.33 (± 0.30) |
| Δ*phoPQ_394_* + *phoP_516_Q_394_* | 516 | 394 | 256-512 | 4-16 | 4-8 | 0.513 (± 0.31) | 0.205 (± 0.18) |
| Δ*phoPQ_394_* + *phoP_394_Q_246_* | 394 | 246 | 256-512 | 64-128 | 8-16 | 39.20 (± 11.60) | 11.33 (±4.80) |
| Δ*phoPQ*_394_ + *phoP_394_Q_516_* | 394 | 516 | 256-512 | 32-64 | 4-8 | 13.44 (± 4.75) | 1.31 (± 0.53) |
| Δ*mgrB_394_* | 394 | 394 | 256->512 | 128-256 | 8 | 49.67 (± 3.86) | 50.67 (± 3.09) |
| Δ*mgrB_394_* + *mgrB*_394_ | 394 | 394 | 256-512 | 4-16 | 4-8 | 0.34 (± 0.24) | 0.22 (± 0.09) |
| Δ*mgrB_394_* + *mgrB*_246(=516)_ | 394 | 394 | 256-512 | 64 | 4-8 | 2.10 (± 1.13) | 0.97 (± 0.66) |

## Supplementary Figure 1: Comparative phylogenomic analysis. To determine the robustness of the clustering of the lineages, in addition to concatenated core gene-based/BAPS clustering (A), phylogenomic grouping was cross-examined by other approaches such as for the core genome by Harvest suite v1.2^4^ (B), and for the whole genome, kSNP3.0^5^ (C) and MASH distance^6^ (D). All the different approaches identically clustered isolates in four lineages as observed by concatenated core gene-based/BAPS clustering. Clade color key: *E. xiangfangensis* L-1: red, L-2: blue, L-3: green, L-4: light green, clades for other species are marked black.


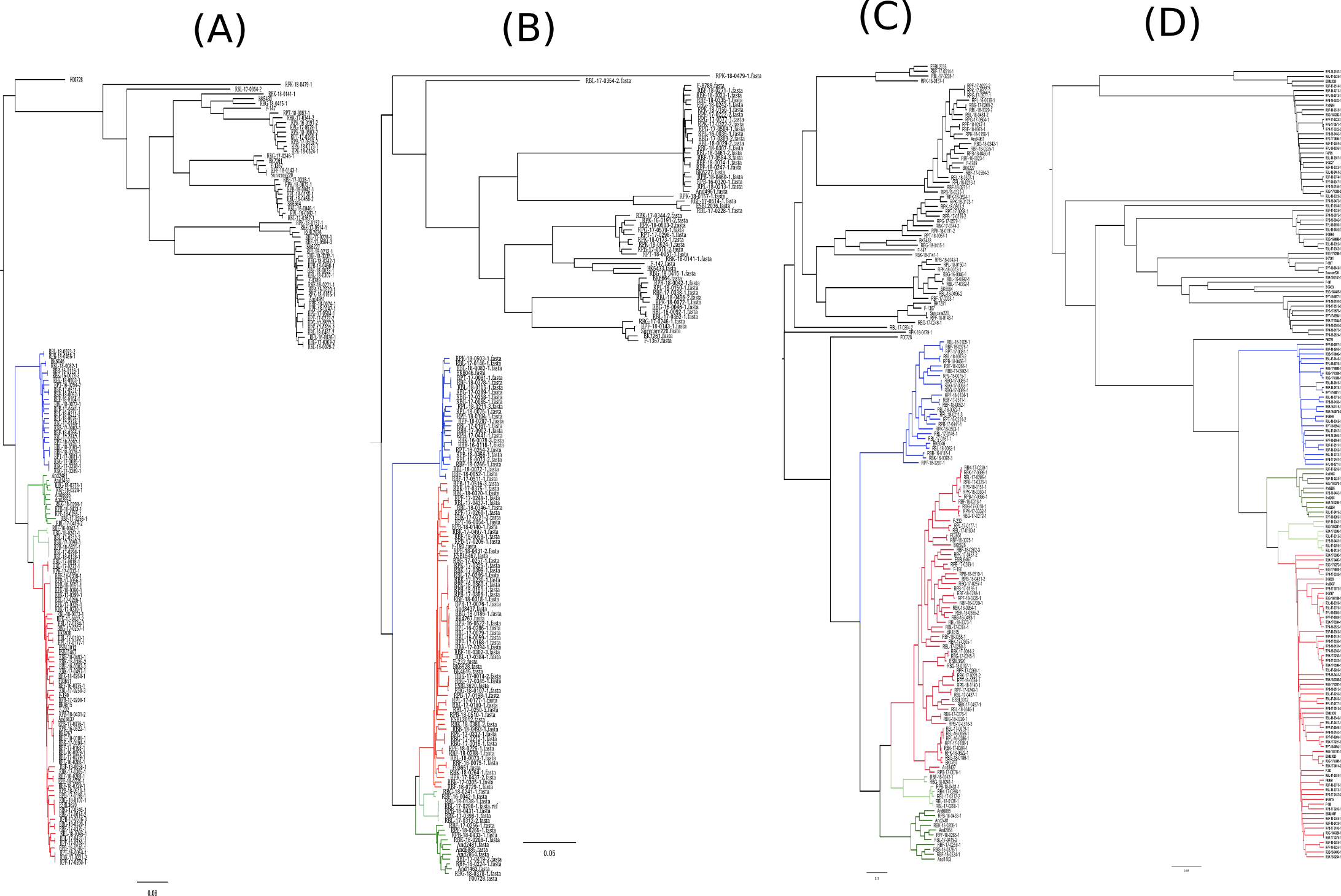


## Supplementary Figure 2: Characteristics of 3246 worldwide *Enterobacter* strains. These 3246 strains were examined for their taxonomic allocation, presence of *mcr-9*, and *arn* gene cassette (*arnBCADTEF*). A total of 68.3% of *Enterobacter* strains were revealed to be *E. xiangfangensis.* These *E. xiangfangensis* were misidentified as *E. cloacae,* *E. hormaechei*, or unidentified *Enterobacter* species in the public databases. Of the *Enterobacter*, isolates of *E. xiangfangensis* L-1 and L-3 (28 and 22%, respectively) were present predominantly. Isolates only of *E. xiangfangensis* L-1 carried the *iro* and *arn* gene cassette (except one sublineage (ST133)). The *arn* gene cassette was absent in other lineages (except ST94 in L-4), while present in other *Enterobacter* species except for the *E. hormaechei* and *E. wuhouensis*. The *arn* gene was absent in the type strain of *E. oligotrophica* and *E. timonensis*. The mobile colistin resistance (*mcr)-9* gene occurred across the worldwide *Enterobacter* species (19.2%) and *E. xiangfangensis* L-1 (30%). The outermost blue triangle shows the phylogenomic location of type strains of 23 *Enterobacter* species (also listed in Supplementary data 2). In the absence of species definition, we propose the abolishment of all the four subspecies. These clusters are now designated as the four lineages of *E. xiangfangensis*. The type strains of *E. hormaechei* subsp. *hormaechei* are included in the phylogenomic cluster of *E. xiangfangensis.*

**
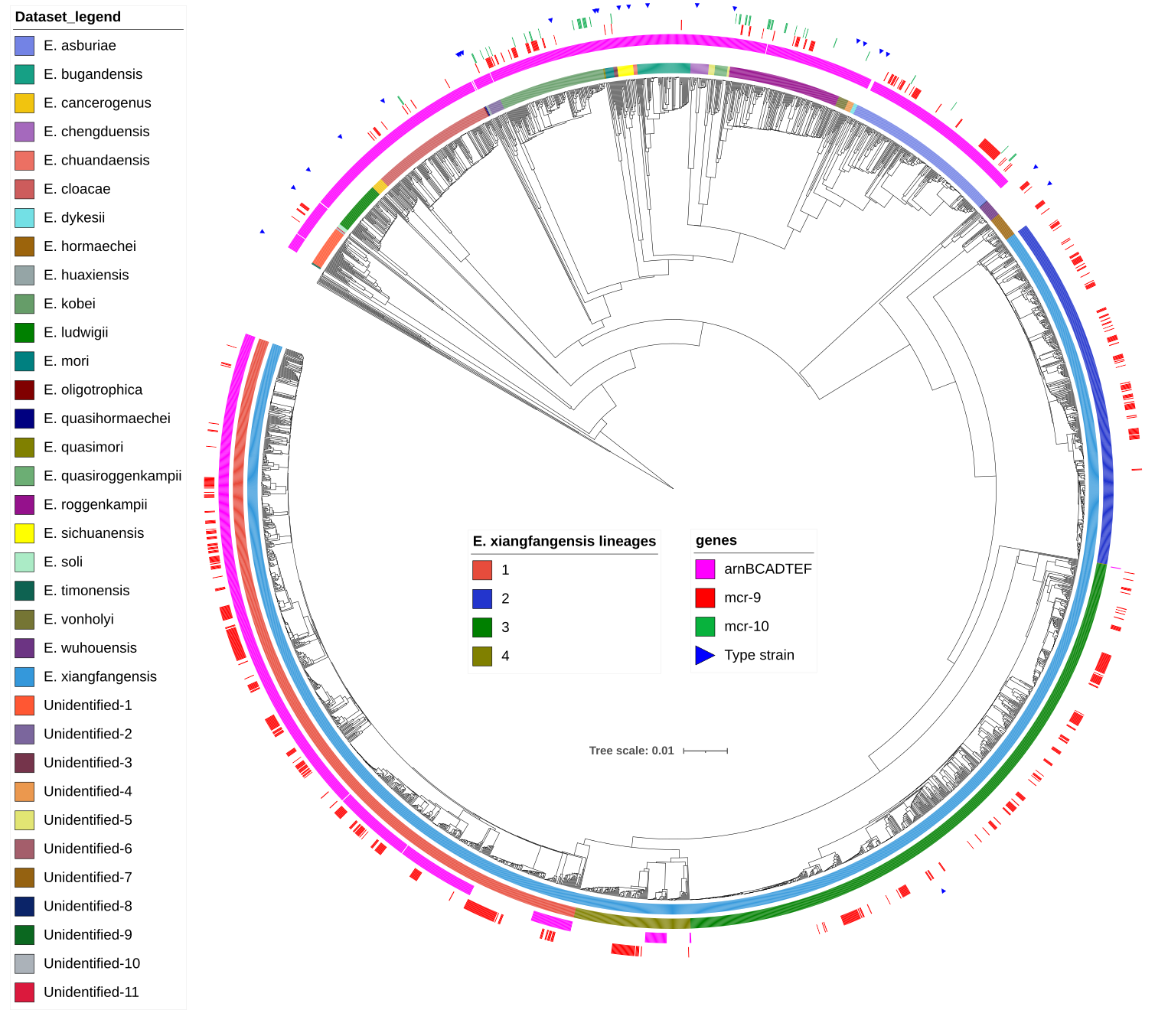
**

## Supplementary Figure 3: Genetic environment of the *mcr-9* gene (shown for nine of the ten completely sequenced genomes). The abbreviation chrom. indicates a chromosomal location; IncHI2, a location on an IncHI2 plasmid; * *E. chengduensis, ^#^ E. ludwigii,* ^$^ truncated *mcr-9.* In the case of one isolate, i.e. *E. xiangfangensis* RPF-17-0260-1, no *mcr-9* gene cassette was detected by closed genome resequencing by Nanopore (Illumina and Nanopore raw reads deposited in the PRJNA622426). Apparently, the IS-associated cassette harboring *mcr-9* gene was lost during the propagation of the culture prepared for resequencing.


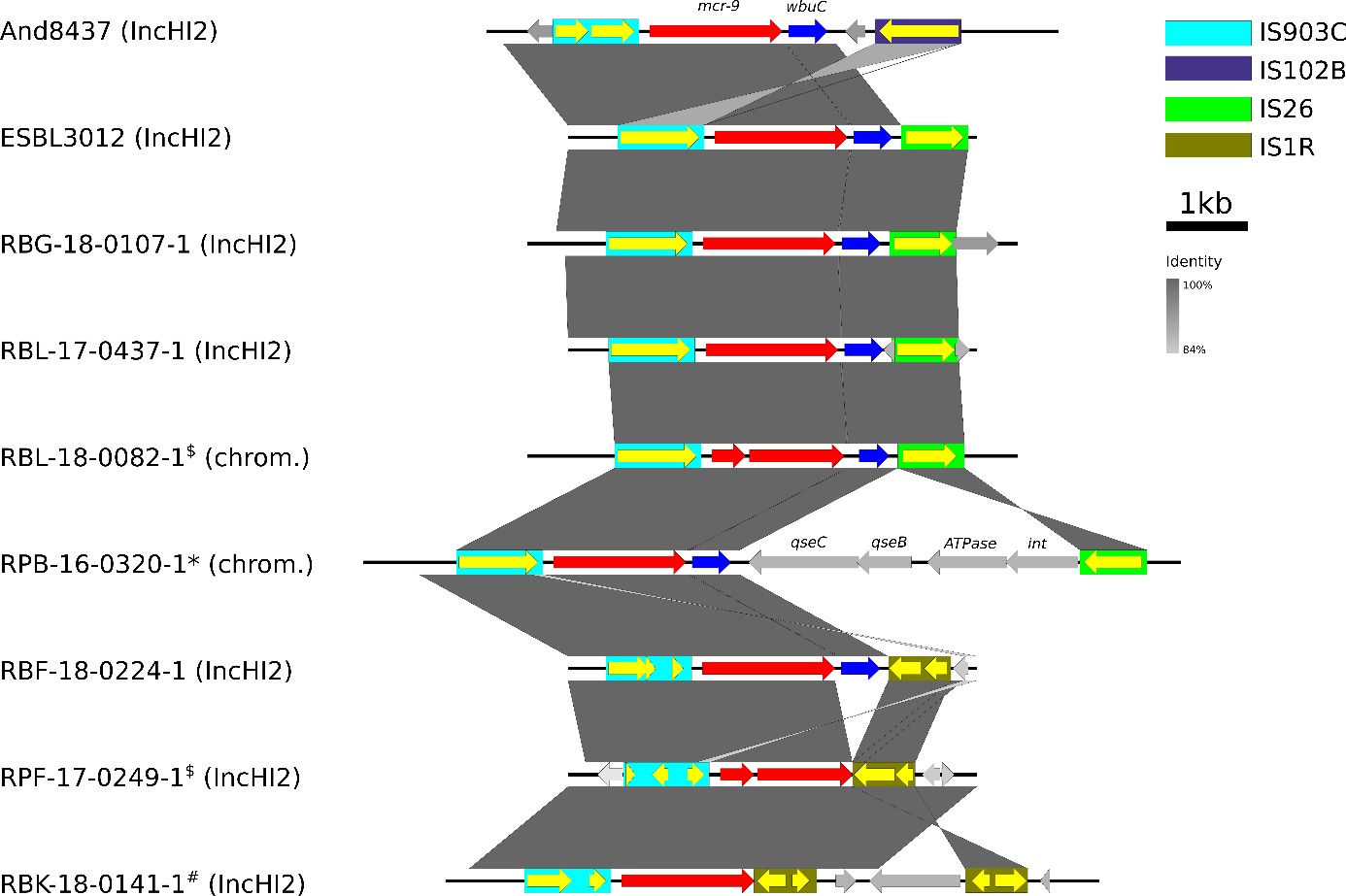


## Supplementary Figure 4: Comparison of the genetic environment of the *arnBCADTEF* gene cassette (*arn*). Except in *E. wuhouensis, E. xiangfangensis* L-2, L-3, and L-4, and ST133 isolates *E. xiangfangensis* L-1, the *arnBCADTEF* gene cassette (*arn*) was present in all *Enterobacter* species. This operon was located in an identical genetic environment (between the gene *padC* and *psf*-1) in all the *Enterobacter* species. The *arn* gene cassette encodes for the transfer of the 4-amino-4-deoxy-L-Arabinose (L-Ara4N) to lipid A phosphate groups at the 1’ and 4’ position.


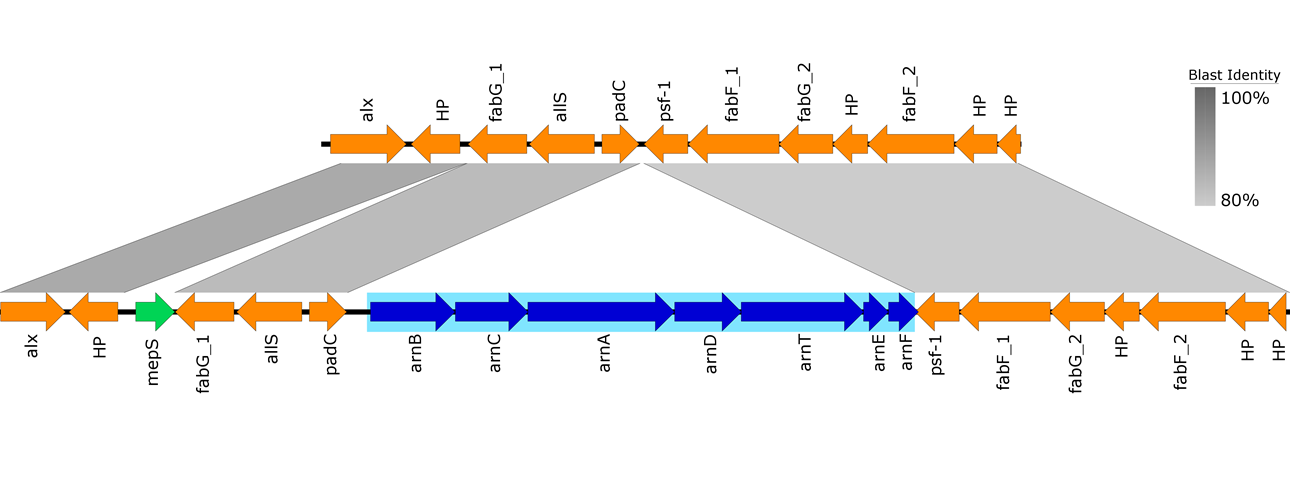


Lineage-1

Lineage-2, 3 and 4

## **Supplementary Figure 5: Mass spectrometric analysis of lipid A isolated from *E. xiangfangensis* L-1 isolates harboring the *arn*-operon grown without colistin or** in presence of 2 mg/L colistin**.** Charge-deconvoluted spectra of representative MS analyses performed in the negative ion mode are shown; the depicted region of 1650–2250 Da comprises the mass range for hexa- and hepta-acylated lipid A species. Presence of colistin in the growth medium leads to a significant increase of L-Ara4N-substitution including double substitution. (Color code: black – lipid A molecules without Ara4N-modification; purple – lipid A molecules with 1 Ara4N; red – lipid A molecules with 2 Ara4N; masses typed in grey and marked with * represent molecules of unknown origin; masses typed in italic style represent sodium adducts (Δ*m* = +21.98 Da) of major abundant lipid A species)

### **(a) *E. xiangfangensis* strain RBK-17-0394-1 [Lineage-1-strain, contains the *arn*-operon**, contains no *mcr-9***]** *top*, lipid A pattern of strain Ex RBK-17-0394-1, if grown without colistin; *bottom*, lipid A pattern of strain Ex RBG-17-0394-1, if grown in presence of 2 mg/L colistin. Mass marked with * represents a penta-acyl lipid A generated by the loss of one fatty acid 3-OH-14:0, mass marked with ^#^ a penta-acyl lipid A generated by the loss of one fatty acid 14:0 from the molecule with 1956.315 Da, respectively.


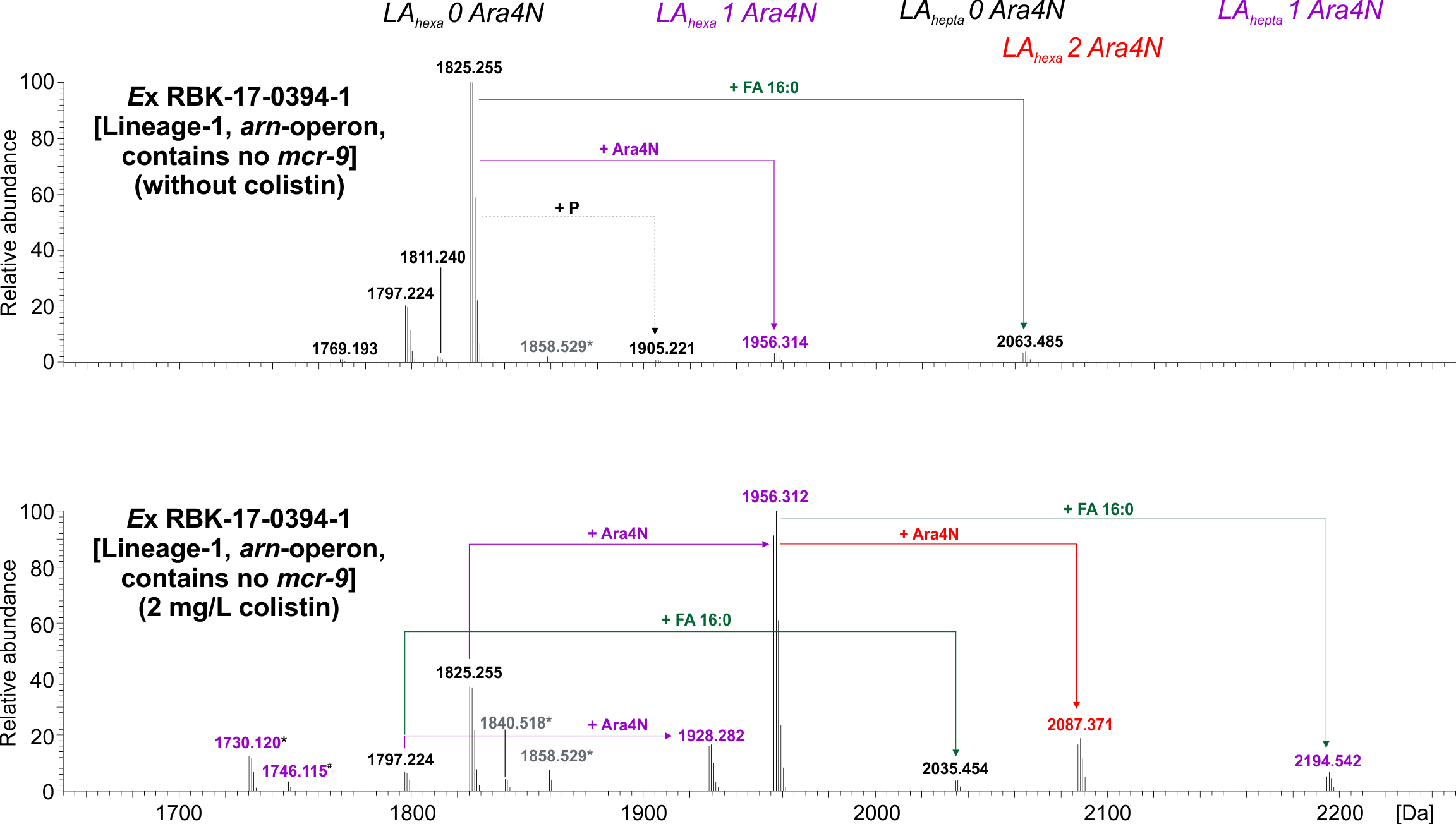


(b) *E. xiangfangensis* strain BK4615 [Lineage-1-strain, contains the *arn*-operon**, contains no *mcr-9***] *top*, lipid A pattern of strain *Ex* BK4615, if grown without colistin; *bottom*, lipid A pattern of strain *Ex* BK4615, if grown in presence of 2 mg/L colistin. Mass marked with * represents a penta-acyl lipid A generated by the loss of one fatty acid 3-OH-14:0 from the molecule with 1956.315 Da, mass labeled with ^$^ represents first isotopic peak.


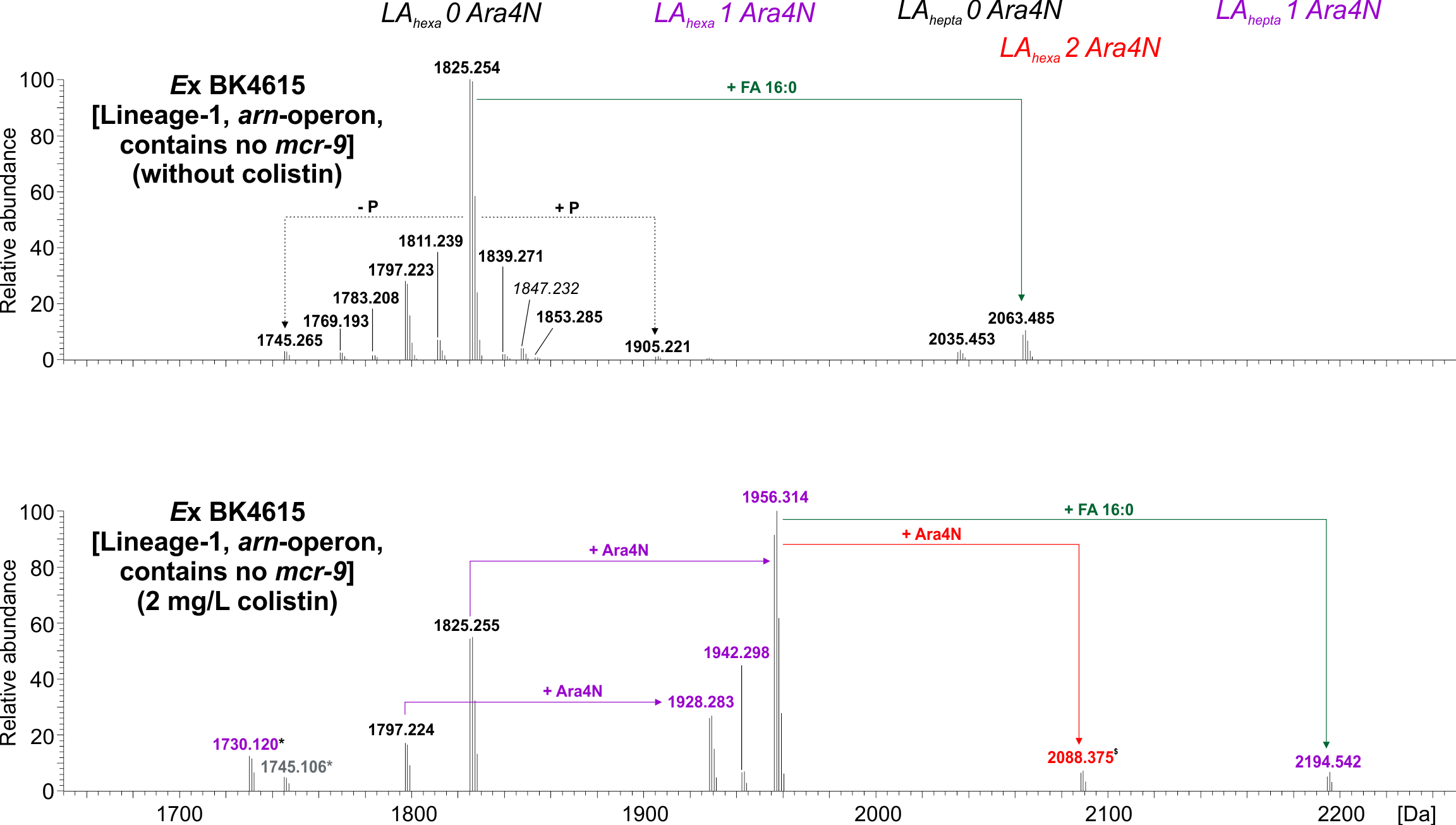


### (c) *E. xiangfangensis* RBG-18-0107-1 [Lineage-1-strain, contains the *arn*-operon*,* contains *mcr-9*]. *top*, lipid A pattern of strain *Ex* RBG-18-0107-1, if grown without colistin; *bottom*, lipid A pattern of strain *Ex* RBG-18-0107-1, if grown in presence of 2 mg/L colistin. Masses marked with * represent penta-acyl lipid A generated by the loss of one fatty acid 3-OH-14:0 from the molecule with 1928.282 Da or 1956.315 Da, respectively. Mass marked with ^#^ represents a penta-acyl lipid A generated by the loss of one fatty acid 14:0 from the molecule with 1956.315 Da.


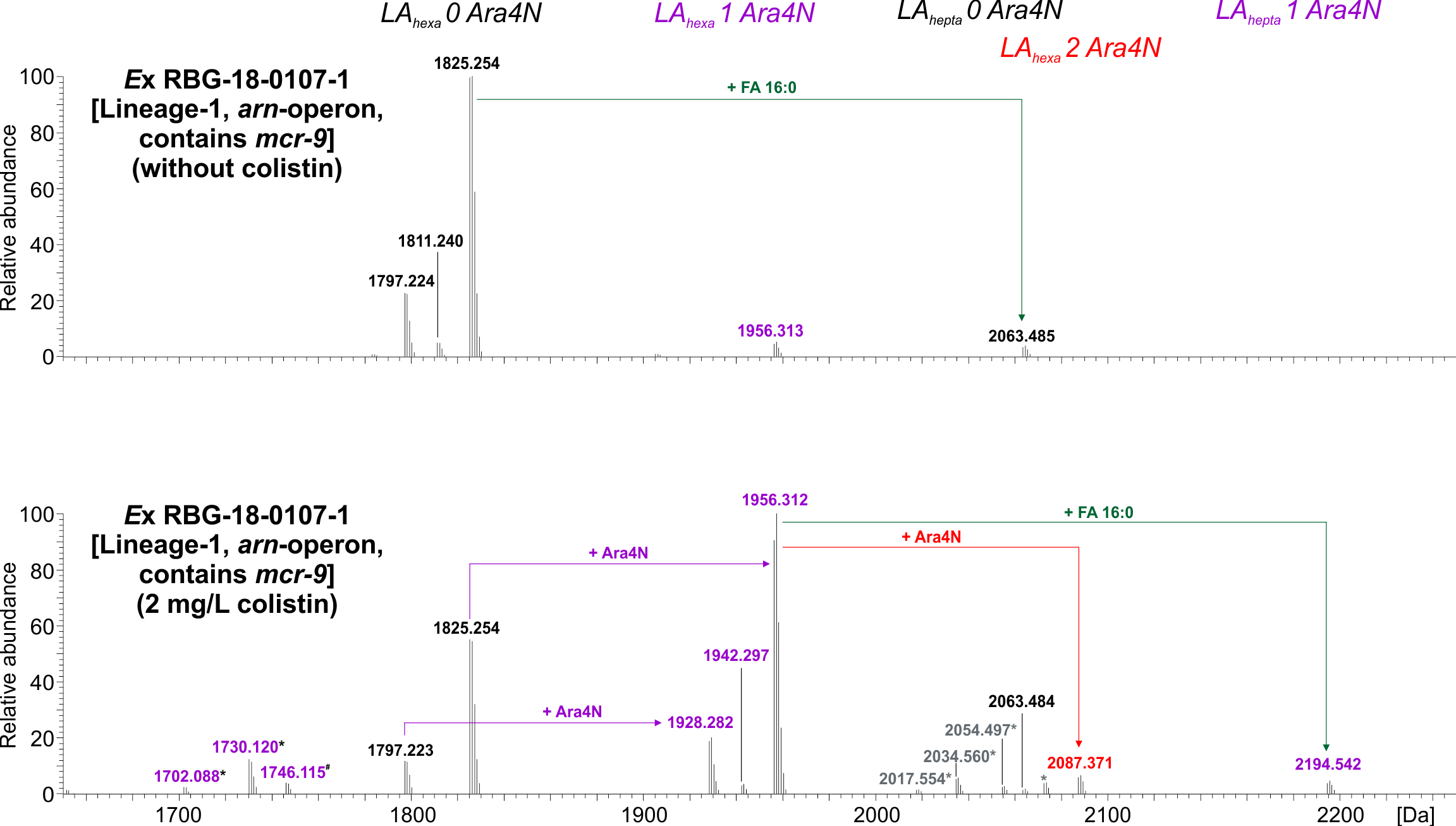


**(d) *E. xiangfangensis* RBL-17-0437-1 [Lineage-1-strain, contains the *arn*-operon, contains *mcr-9*].** *top*, lipid A pattern of strain *Ex* RBL-17-0437-1, if grown without colistin; *bottom*, lipid A pattern of strain *Ex* RBL-17-0437-1, if grown in the presence of 2 mg/L colistin. Mass marked with * represents a penta-acyl lipid A generated by the loss of one fatty acid 3-OH-14:0, mass marked with # represents a penta-acyl lipid A generated by the loss of one fatty acid 14:0 from the molecule with 1956.315 Da, respectively.


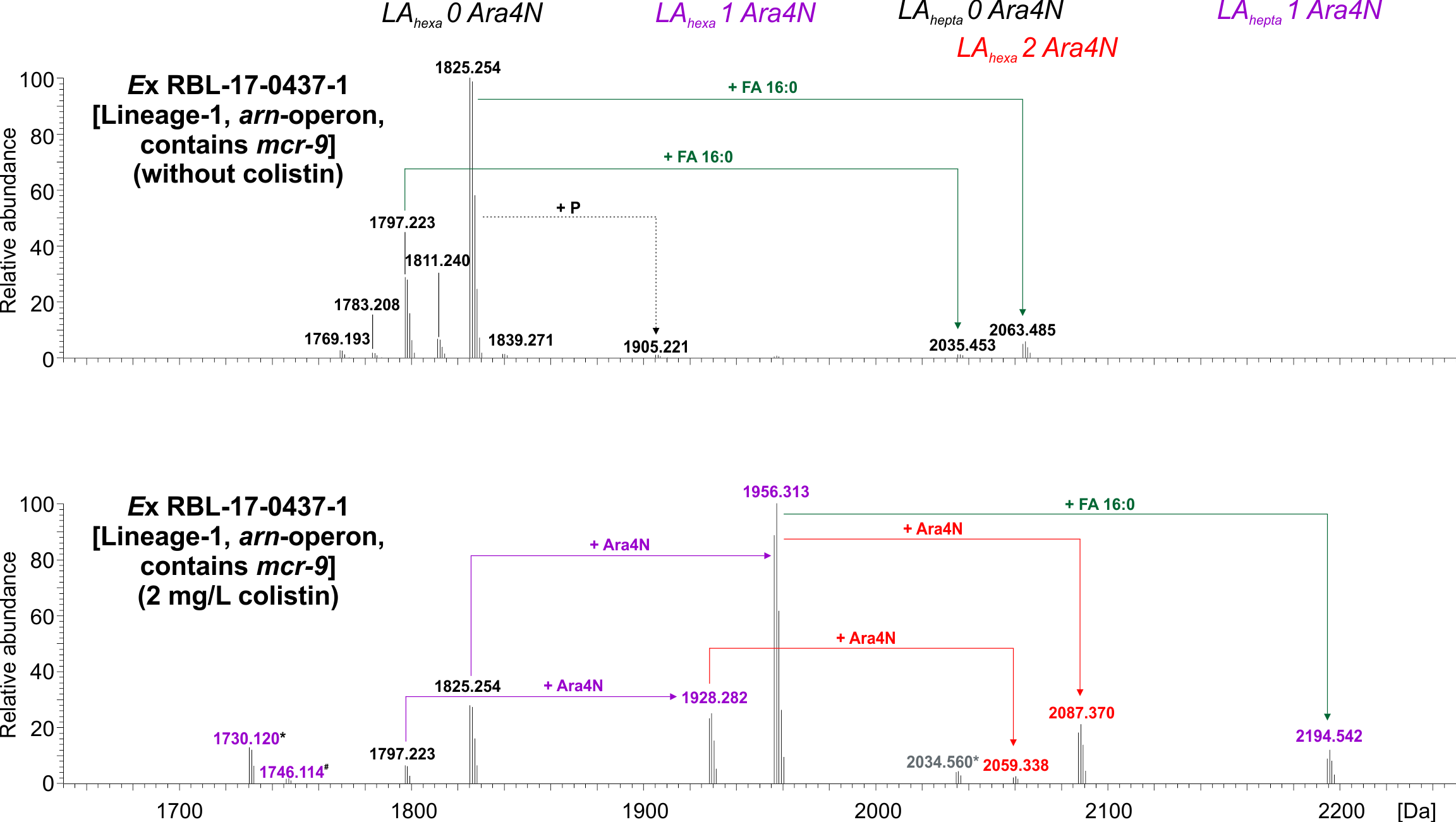


**(e) *E. xiangfangensis* RBL-17-0260-1 [Lineage-1-strain, contains the *arn*-operon, contains *mcr-9*].** *top*, lipid A pattern of strain *Ex* RBL-17-0260-1, if grown without colistin; *bottom*, lipid A pattern of strain *Ex* RBL-17-0260-1, if grown in presence of 2 mg/L colistin.


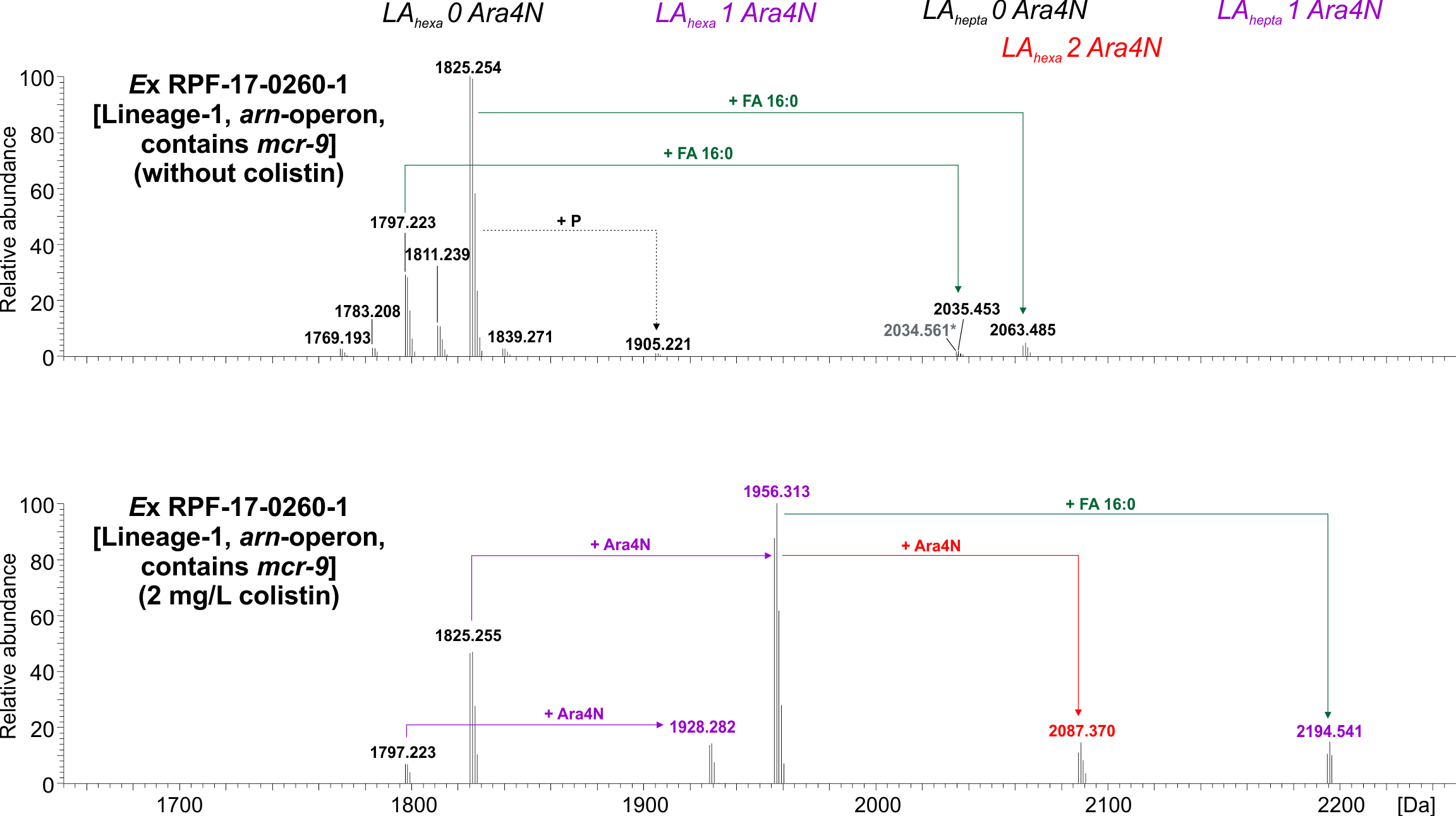


## Supplementary Figure 6: LIVE/DEAD^TM^ staining assay for detecting heteroresistance. The PAP test revealed that only a portion of an isogenic growing population survives when exposed to colistin. Using the LIVE/DEAD^TM^ staining, we established a rapid short-term assay to monitor levels of resistance toward colistin. In brief, colistin treatment was carried out (for *E. xiangfangensis* L-1 RBK-17-0394-1 (WT) and mutant strains) by exposing cells to 64 mg/L colistin sulphate for 15 min. Control was maintained by exposing cells to 0.85% NaCl. Treated and control cells were stained with LIVE/DEAD™ BacLight™ that stains live cells in green (SYTO9 DNA stain, can penetrate intact cell membrane) and dead or membrane-compromised cells in orange-red (Propidium iodide DNA stain, can penetrate only when the membrane integrity is compromised). (a) Colistin treated and untreated cells. Colistin treatment resulted in the survival of only 11.81% of population of WT. Cells of Δ*arnBCADTEF* or Δ*phoPQ* mutants that do not exhibit modified forms of lipid A with L-Ara4N do not survive (survival rate <5%). In the case of Δ*mgrB* mutant, where the *phoPQ/arnBCADTEF* axis is functional, the highest possible modification of lipid A with L-Ara4N is detected and showed the highest percentage of surviving bacteria despite colistin treatment (survival rate 85.25%). (b) Evaluation of cell membrane integrity: Bacteria treated with colistin were stained with FM4-64 (stain cell membrane in red) and DAPI (blue DNA stain, passes less efficiently through intact cell membrane). WT cells are variably stained with blue stain indicating the degree of membranes with loss of integrity. No blue stain could be observed in Δ*mgrB* cells showing that the population comprises of bacteria with an intact membrane. All the Δ*arnBCADTEF* and Δ*phoPQ* cells strongly stain in blue implying heavily compromised cell membranes as a result of colistin exposure. Scale bar: 5 μm. The red and green fluorescence images were acquired under Plan Apochromat 60x Oil immersion lens (Keyence Biozero BZ-8000K) and images were merged by default BZ analyzer v3.61 settings.

| **A** |  |  |  |
| --- | --- | --- | --- |
| WT untreated | Δ*mgrB* untreated | Δ*arnBCADTEF* untreated | Δ*phoPQ* untreated |
| 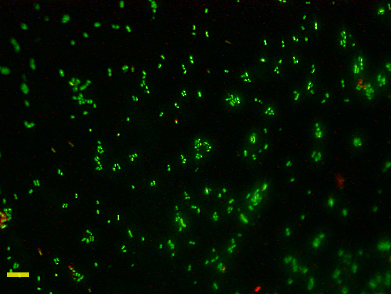 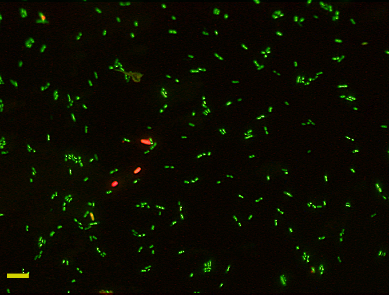 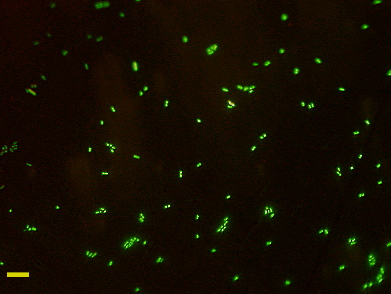 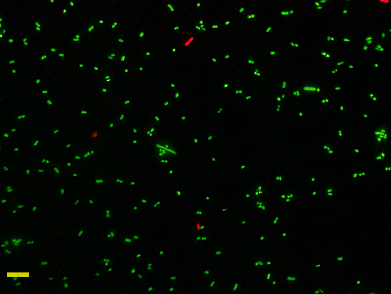 | | | |
|  |  |  |  |
| WT treated | Δ*mgrB* treated | Δ*arnBCADTEF* treated | Δ*phoPQ* treated |
| 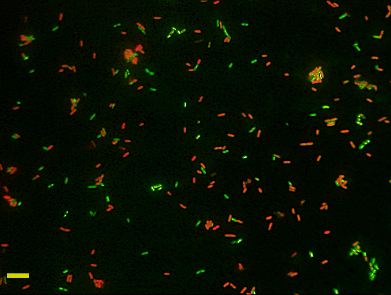 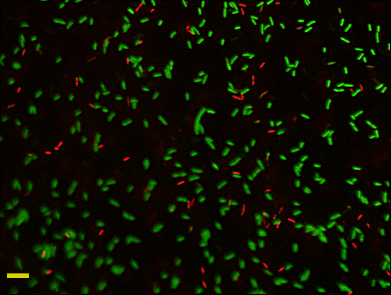 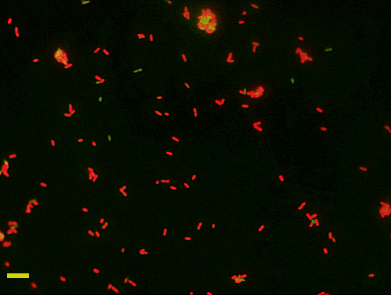 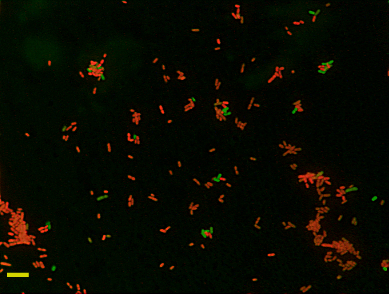 | | | |
|  |  |  |  |
| **B** |  |  |  |
| WT treated | Δ*mgrB* treated | Δ*arnBCADTEF* treated | Δ*phoPQ* treated |
| 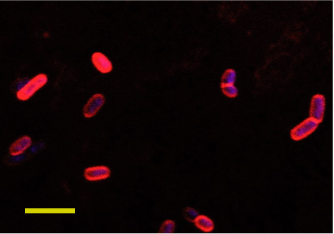 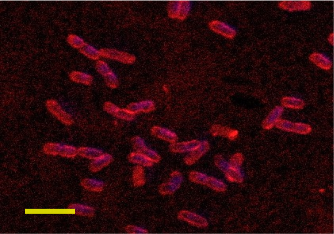 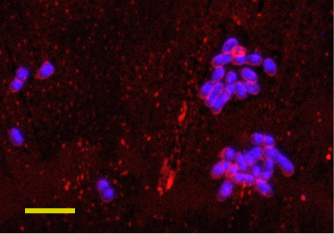 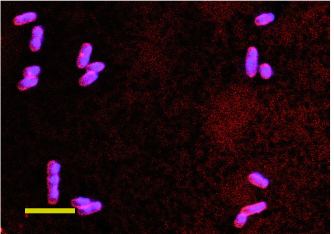 | | | |

## Supplementary Figure 7: LiveDead^TM^ staining of the WT (Ex394), Ex394Δ*phoPQ*::*phoPQ_394_*_,_ Ex394Δ*phoPQ*::*phoPQ_246_*_,_ Ex394Δ*phoPQ*::*phoPQ_516_*_,_ before and after colistin treatment. WT and complement mutants (Ex394Δ*phoPQ*::*phoPQ_394_*) treated with the colistin showed 11.81 and 47.43% of cell survival, respectively. The Ex394Δ*phoPQ*::*phoPQ_246_* and Ex394Δ*phoPQ*::*phoPQ_516_* showed 79.74 and 72.92% of cell survival, respectively. The colistin treatment was carried out by exposing cells to 64 mg/L colistin sulphate for 15 min, followed by staining using LIVE/DEAD™ BacLight™. Scale bar: 5 μm.

| WT untreated | Δ*phoPQ* + Δ*phoPQ_394_* untreated | Δ*phoPQ* + Δ*phoPQ_246_* untreated | Δ*phoPQ* + Δ*phoPQ_516_* untreated |
| --- | --- | --- | --- |
| 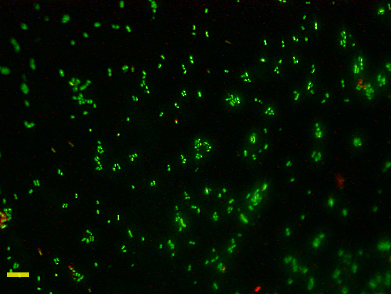 | 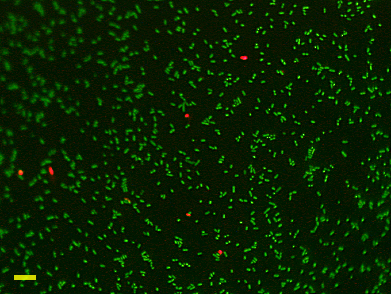 | 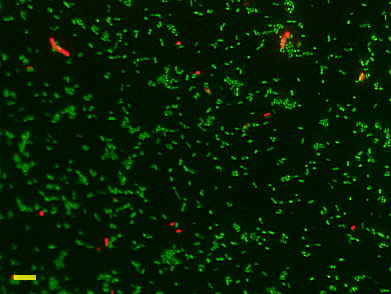 | 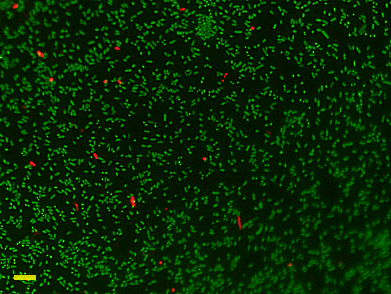 |
| WT treated | Δ*phoPQ* + Δ*phoPQ_394_* treated | Δ*phoPQ* + Δ*phoPQ_246_* treated | Δ*phoPQ* + Δ*phoPQ_516_* treated |
| 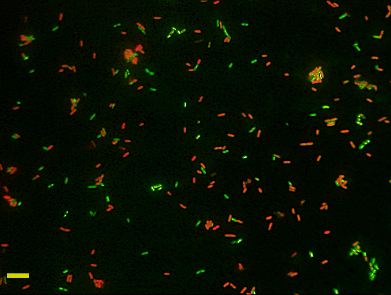 | 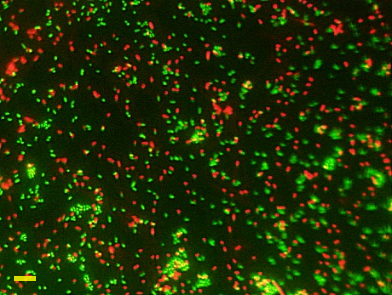 | 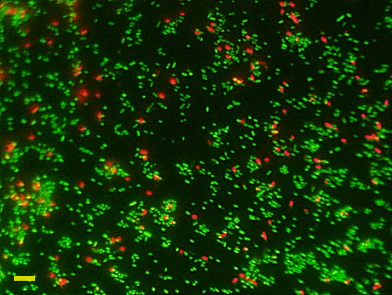 | 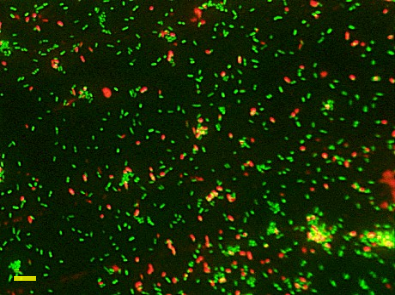 |

## **Supplementary Figure 8: The relative** gene expression of the *arnBCADTEF* (*arn*), *phoPQ* and *mgrB* genes in Ex394Δ*phoPQ* mutant complemented with *phoPQ_246_* and *phoPQ_516_*. The Ex394Δ*phoPQ* strain complemented with *phoPQ* from other species. viz., *E. bugandensis* (*phoPQ_246_*) and *E. roggenkampii* (*phoPQ_516_*) resulted in higher expression of the *arnBCADTEF.* The Ex394Δ*phoPQ* complemented with the original *phoPQ* (*phoPQ_394_*) resulted in restoring original MIC_LB_ (8-32 mg/L) with skip-well phenomenon in broth microdilution assay, and heteroresistance frequency (0.63% (± 0.09)) in PAP test. Complementation of Ex394Δ*phoPQ* with *phoPQ_246_* and *phoPQ_516_*, in both the cases, resulted in the MIC_LB_ of 32 mg/L without any skip-well phenomenon. In the case of PAP test heteroresistance frequency for the Ex394Δ*phoPQ* + *phoPQ_246_* was 42.33% (± 4.78), while Ex394Δ*phoPQ* + *phoPQ_516_* showed 10.83% (± 1.43). In the case of expression of *phoPQ* and *mgrB*, no significant changes were observed in both the compliments. Mean and standard deviation values for three independent experiments, each comprising of three technical replicates, are presented.

## Supplementary Figure 9: **Interaction between the PhoQ and MgrB.** The interaction models between PhoQ and MgrB were predicted *ab initio* using RoseTTAFold and HADDOCK 2.4, respectively. While the periplasmic region of both proteins aligned to each other inferring their interactions, the α/β-core (α4/α5 and β3/β5/β6) and the acidic patch of PhoQ were observed to closely interact with the periplasmic region of the MgrB (a) and (b): dimer predicted for PhoQ (a) and MgrB (b) by RoseTTAFold. (c) Interactions of α/β-core of PhoQ and MgrB regions. (d) detailed profiles of periplasmic domain interactions between the MgrB and PhoQ variants in different mutants.


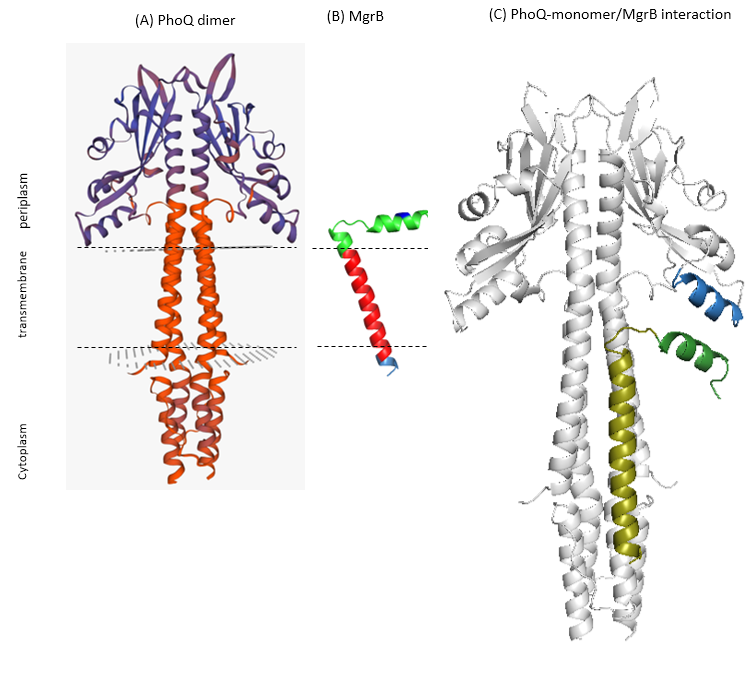


(d)


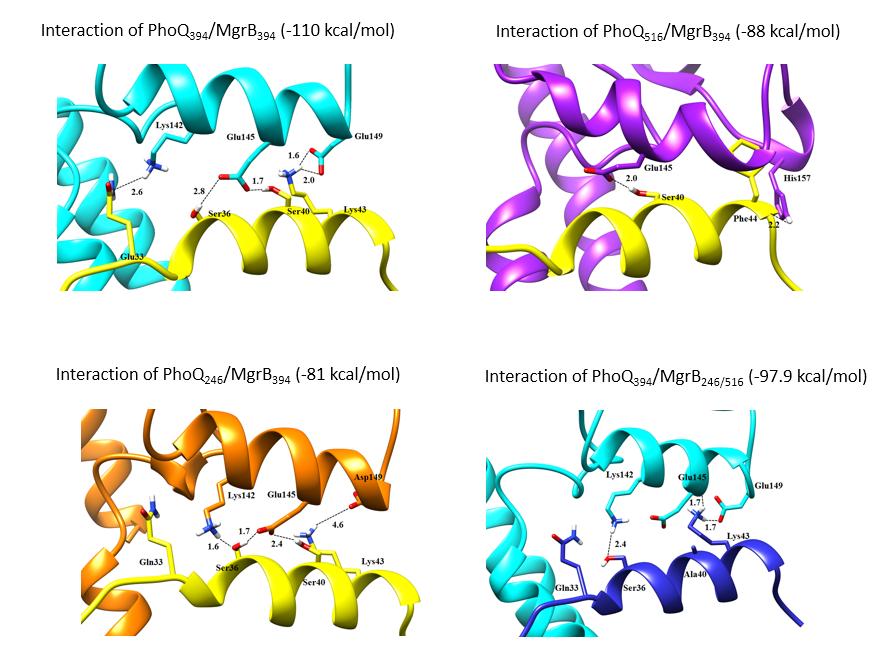


# Supplementary data

## Supplementary data 1: (a) Details of the contemporary (year 2016-2018) and historical (year 2011-2015) *Enterobacter* isolates collected and their antibiotic resistance phenotype, (b) metadata of *Enterobacter* isolates considered in this study. The sheet list isolate’ source, genome-based identification of the species, profiles of the mcr-9 gene, *arnBCADTEF* gene cassette and ecr gene, hsp60, MLST types, lineage details, HR frequency, MICs ( EUCAST recommended protocol, and LB medium by higher initial inoculum), (c) Illumina- and (d) nanopore-sequencing statistics.

## Supplementary data 2: Details of the 3246 worldwide *Enterobacter* isolates considered for the study.

## Supplementary data 3: Details of the strain and primers.

## Supplementary data 4: Mass spectrometric details of Lipid-A profiles of *Enterobacter* species when grown in the presence or absence of colistin*.*

## Supplementary data 5: Historical details of the *Enterobacter* taxonomy. A total of 51 species have been classified under the genus *Enterobacter.* With the evolution of the techniques used for the classification of the prokaryotes, number of species shifted to return to the *Enterobacter* genus. Based on OGRI, we determined 23 species to be valid (as of March 2022). Details of each taxonomic shift is listed in this table.

## Supplementary data 6: Mutations in the *phoP*, *phoQ*, and the *arn* promoter region of studied *Enterobacter* isolates. Altered amino acids in *phoP* (a), *phoQ* (b), and nucleotide sequenced of the *arn* promoter region (-10 and -35 as per Kang et al., 2020)^7^ (c). In the case of the *phoQ* gene, at a total of 94 of 488 (19.2%) locations, amino acids got substituted (non-synonymous mutation). The majority of the alterations were present in the sensor (26%, 39/150 AA) and histidine kinase domains (18.9%, 42/222 AA). While *E. xiangfangensis* showed relatively low MIC_LB_ (between 2 to 64 mg/L) and low heteroresistance frequency in the PAP test, other *Enterobacter* species carrying functional *phoPQ*/*arnBCADTEF* gene cassette showed higher MIC_LB_ (8 to >128 mg/L) and higher heteroresistance frequency. Key: PAP: Population analysis profile phenotype measuring the degree of heteroresistance.

## Supplementary data 7: Single nucleotide differences among the isolates. A total of 52 isolates clustered in 20 phylogenomic clades that could not be distinguished further. The SNVs were derived by mapping filtered reads against the closed genome of ESBL3012 using the snippy tool.

# Supplementary references:

1. Cho, G. S. *et al.* Polyphasic study of antibiotic-resistant enterobacteria isolated from fresh produce in Germany and description of Enterobacter vonholyi sp. nov. isolated from marjoram and Enterobacter dykesii sp. nov. isolated from mung bean sprout. *Syst. Appl. Microbiol.* **44**, 126174 (2021).

2. Sutton, G. G., Brinkac, L. M., Clark, T. H. & Fouts, D. E. Enterobacter hormaechei subsp. hoffmannii subsp. nov., Enterobacter hormaechei subsp. xiangfangensis comb. nov., Enterobacter roggenkampii sp. nov., and Enterobacter muelleri is a later heterotypic synonym of Enterobacter asburiae based on computational a. *F1000Research* **7**, 521 (2018).

3. Chun, J. *et al.* Proposed minimal standards for the use of genome data for the taxonomy of prokaryotes. *Int. J. Syst. Evol. Microbiol.* **68**, 461–466 (2018).

4. Treangen, T. J., Ondov, B. D., Koren, S. & Phillippy, A. M. The Harvest suite for rapid core-genome alignment and visualization of thousands of intraspecific microbial genomes. *Genome Biol.* **15**, 524 (2014).

5. Gardner, S. N., Slezak, T. & Hall, B. G. kSNP3.0: SNP detection and phylogenetic analysis of genomes without genome alignment or reference genome. *Bioinformatics* **31**, (2015).

6. Ondov, B. D. *et al.* Mash: fast genome and metagenome distance estimation using MinHash. *Genome Biol.* **17**, 132 (2016).

7. Kang, K. N. *et al.* Colistin heteroresistance in Enterobacter cloacae is regulated by PhoPQ-dependent 4-amino-4-deoxy-l-arabinose addition to lipid A. *Mol. Microbiol.* **111**, 1604–1616 (2019).
